# Mitogen-activated protein kinase mediates mevalonate-stimulated human mesangial cell proliferation

**DOI:** 10.3892/mmr.2015.3715

**Published:** 2015-05-04

**Authors:** XIAOSHUANG ZHOU, CHEN WANG, JIHUA TIAN, YANHONG WANG, YAFENG LI, ZHAOYONG HU, RONGSHAN LI

**Affiliations:** 1 Department of Nephrology, Provincial People’s Hospital of Shanxi Medical University, Shanxi Provincial People’s Hospital, Shanxi Kidney Disease Institute, Taiyuan, Shanxi 030001, P.R. China; 2 Department of Pathology, The Second Hospital of Shanxi Medical University, Taiyuan, Shanxi 030001, P.R. China; 3 Department of Microbiology and Immunology, Shanxi Medical University, Taiyuan, Shanxi 030001, P.R. China; 4 Department of Nephrology, Baylor College of Medicine, Houston, TX 77030, USA

**Keywords:** 3-hydroxy-3-methylglutaryl coenzyme A reductase inhibitor, mevalonate, apoptosis, mesangial cell

## Abstract

The metabolic products of intracellular mevalonate (MVA) are important for the growth of eukaryotic cells. These products include cholesterol and several non-sterol isoprenoids. It has been reported that 3-hydroxy-3-methylglutaryl coenzyme A reductase inhibitors ameliorate glomerular injury in several experimental models of progressive glomerular disease by inhibiting the production of MVA and its metabolites. However, the mechanisms by which MVA stimulates the growth of human mesangial cells (HMCs) remain to be elucidated. To investigate the role of MVA in HMC proliferation, apoptosis, cell cycle and accumulation of extracellular matrix (ECM), the effects of MVA on HMCs at different durations and at various doses were evaluated. To examine the mechanisms of the effects of MVA on HMCs, the cells were treated with MVA, with or without PD98059, an extracellular signal-regulated kinase (ERK) inhibitor, SP600125, c-Jun NH2-teminal kinase (JNK) inhibitor, or SB203580, a P38 mitogen-activated protein kinase (MAPK) inhibitor. A 3-(4,5-dimethylthiazol-2-yl)-2,5-diphenyl tetrazolium bromide reduction assay was used to measure the proliferation of the HMCs, a flow cytometric assay was used to assess the proliferative index, and an ELISA was performed to determine the expression of transforming growth factor-β1 (TGF-β1), Type IV and Type I collagen (Col-IV and Col-I). The expression of B-cell lymphoma 2 (Bcl-2), Bcl-2-associated X protein (Bax), phosphorylated (p)-ERK1/2, p-JNK and p-p38 were also examined using western blot analysis. MVA significantly stimulated HMC proliferation and markedly increased the secretion of TGF-β1 and expression levels of Col-IV and Col-I. In addition, treatment with MVA significantly upregulated the expression of Bcl-2 and suppressed the expression of Bax in the HMCs. These responses were partially inhibited by the addition of inhibitors of ERK or JNK, however, they were not inhibited by the p38 MAPK inhibitor. These results demonstrated that MVA promoted HMC proliferation and ECM protein expression, which were associated with an increase in the expression of TGF-β1 and the inhibition of apoptosis. These effects were mediated, at least in part, by the JNK and ERK pathways.

## Introduction

Mesangial proliferative glomerulonephritis is the most common type of primary glomerular disease in China. It is characterized by the proliferation of mesangial cells (MCs) and deposition of extracellular matrix (ECM), which results in glomerular sclerosis, and end-stage renal disease (1). MCs are involved in various types of glomerular injury via the proliferation and secretion of cytokines, including transforming growth factor-β (TGF-β). TGF-β stimulates the expression of ECM proteins, including collagen type IV (col-IV) and interstitial collagen, including collagen type I (col-I) (2). The dysregulation of cell apoptosis also contributes to the proliferation of MCs and ECM deposition (3). B-cell lymphoma 2 (Bcl-2) family members, including the Bcl-2 anti-apoptotic and Bcl-2-associated X protein (Bax) a pro-apoptotic proteins, are important regulators of cell apoptosis (4). However, whether these apoptotic proteins are involved in MCA-stimulated MC proliferation remains to be elucidated.

Hyperlipemia is responsible for a number of renal diseases (5), and the 3-hydroxy-3-methylglutaryl coenzyme A (HMG-CoA) reductase inhibitors exert modulatory effects on a number of cell signaling cascades by preventing the synthesis of various isoprenoids derived from the mevalonate (MVA) pathway (6). Mitogen-activated protein kinases (MAPKs), including extracellular signal-regulated kinase (ERK), c-Jun NH2-teminal kinase (JNK)/stress-activated protein kinase, (SAPK), P38 MAPK and ERK5/big MAPK 1 (BMK1) are key regulators of MC proliferation and ECM deposition, and are thus closely associated with the development of mesangial proliferative glomerulonephritis (7,8). However, the effect of MVA on MCs, its underlying mechanisms, its effect on MAPKs and downstream transcription factors and the association between MAPKs and MCs remain to be elucidated. The aim of the present study was to investigate the effects of MVA on human mesangial cell (HMC) proliferation, apoptosis, cell cycle and ECM deposition, as well as the role of TGF-β1 and the MAPKs in the process, in order to examine the mechanism of MVA in the development of mesangial proliferative glomerulonephritis.

## Materials and methods

### HMC culture

The T-SV40 HMC cell line was provided by Dr Li Xuewang (Peking Union Medical College Hospital, Beijing, China). The cells were routinely maintained in RPMI-1640 (Sigma-Aldrich, St. Louis, MO, USA), containing 10% fetal calf serum (FCS; Sijiqing Biological Engineering Materials Co., Ltd., Hangzhou, China) and supplemented with 100 U/ml penicillin and 100 *μ*g/ml streptomycin (Sijiqing Biological Engineering Materials Co., Ltd.) at 37°C. The culture medium was replaced every 2 days. When the cells reached confluence, they were subcultured at a ratio of 1:4, using the same incubation medium.

### Experimental design

The HMCs (60% confluent) were trypsinized (Sijiqing Biological Engineering Materials Co., Ltd.) and seeded (4×10^4^ cells/cm^2^) into petri dishes at 37°C and were cultured with MVA (Sigma-Aldrich) at various concentrations (0, 10^−9^, 10^−8^, 10^−7^, 10^−6^, 10^−5^, 10^−4^ and 10^−3^ M) for 24 h, and at 10^−7^ M for 12, 24 or 48 h, to evaluate the effects of dose and time on HMC proliferation.

The HMCs (1×10^5^ cells/ml) were then cultured with MVA (1×10^−7^ mol/l), either alone or in the presence of 50 *μ*mol/l PD98059, an ERK inhibitor; 50 *μ*mol/l SP600125, a JNK inhibitor or 50 *μ*mol/l SB203580, a P38 MAPK inhibitor (all Axxora-Boppard, Shanghai, China). The cells were cultured for 24 h at 37°C with RPMI-1640 supplemented with 10% FCS and were then serum-starved for 24 h. The cells were centrifuged at 500 x g for 5 min at 25°C, the supernatants were then collected and the total protein was extracted from the cells. A 3-(4,5-dimethylthiazol-2-yl)-2,5-diphenyl tetrazolium bromide (MTT) reduction assay was performed to measure the proliferation of the HMCs. A flow cytometric assay was performed to assess the proliferative index (PI) and an ELISA was performed to determine the secretion of TGF-β1, Col-IV and Col-I. The expression levels of Bcl-2 and Bax were analyzed using western blot analysis. In certain experiments, p-ERK1/2, p-JNK and p-p38 were measured using western blot analysis.

### MTT assay

The number of living cells in the HMC cultures was assayed using an MTT assay (Sigma-Aldrich). The MTT-formazan product was dissolved in phosphate-buffered saline (PBS; Sigma-Aldrich). Following the addition of RPMI-1640 medium containing 10% MTT, the cells (1×10^5^ cells/ml) were incubated at 37°C for 4 h, the medium was aspirated and the cells were lysed by the addition of 100 *μ*l dimethyl sulfoxide (DMSO; Sigma-Aldrich). Subsequently, 10 *μ*lof each sample was diluted in 90 *μ*l fresh DMSO. The samples were then mixed on a mechanical plate mixer, and the optical density of each sample at the test and reference wavelengths of 550 and 650 nm, respectively was measured using a microplate-reader (Model 550; Bio-Rad Laboratories, Inc., Hercules, CA, USA).

### Analysis of PI and apoptosis using flow cytometry

The HMCs were cultured in 10-cm dishes (1×10^5^ cells/ml) RPMI-1640 medium containing 10% FCS and then incubated for 24 h in 10% FCS. The cells were collected and fixed with 1% methanol-free formaldehyde (Sigma-Aldrich) for 20 min. The samples were washed twice with PBS and then resuspended in 70% ethanol (Sijiqing Biological Engineering Materials Co., Ltd.) for 1 h at 4°C. The fixed and permeated cells were collected by centrifugation at 500 x g for 5 min at 25°C, washed with PBS and incubated with RNase (50 *μ*g/ml; Sigma-Aldrich) for 10 min at room temperature. Finally, 200 *μ*l propidium iodide (65 *μ*g/ml; Sigma-Aldrich) was added to each dish for 10 min on ice, in order to stain the nuclei. The numbers of cells in the G1/S, and G2/M phases of the cell cycle were the analyzed using flow cytometry (BD Accuri™ C6; BD Biosciences, San Jose, CA, USA). The above experiments were repeated six times. Each sample was analyzed using CellQuest version 3.0 software (BD Biosciences) to assess the percentage of cells in each phase of the cell cycle and the apoptotic rate. The PI was calculated according to the following equation: PI = (S + G2/M) / (G0/G1 + S + G2/M).

### Measurement of TGF-β1, Col-I and Col-IV using ELISA

The culture supernatant (250 *μ*l) was removed from each well and incubated with 5 *μ*l 1 N HCl for 60 min to activate the latent TGF-β1, Col-I or Col-IV. Following neutralization of the supernatants with 1 N NaOH, the samples were analyzed using a commercial human TGF-β1, Col-I or Col-IV ELISA kit (Promega, Madison, WI, USA), according to the manufacturer’s instructions. A standard curve was constructed using serial dilutions of ultrapure human TGF-β1, Col-I or Col-IV (Promega). The cells were harvested by trypsinization and counted using a microscope (BX51; Olympus Corporation, Tokyo, Japan). Each sample was measured in duplicate.

### Western blot analysis of the expression levels of Bcl-2, Bax, p-ERK1/2, p-JNK and p-p38

The cells (1×10^5^ cells/ml) were homogenized in radioimmunoprecipitation assay buffer (Thermo Fisher Biochemical, Shanghai, China). The homogenates were centrifuged at 12,000 g for 10 min at 4°C, and the supernatants were collected. The total fractions were denatured in sample buffer [20 *μ*l protein samples + 5 *μ*l 5X loading buffer (Sigma-Aldrich)] at 100°C for 5 min. The protein (60 mg) was electrophoresed on 10% SDS-polyacrylamide gels, then transferred onto nitrocellulose membranes (Merck Millipore, Shanghai, China), blocked with 5% non-fat milk in Tris-buffered saline with 0.05% Tween 20 (TBST) buffer overnight at 4°C. The membranes were then incubated with mouse monoclonal anti-Bax (1:1,000; cat. no. ab5714; Abcam, Cambridge, UK), mouse monoclonal anti-Bcl-2 (1:1,000; cat. no. ab201566; Abcam), rabbit monoclonal anti-p-p38 MAPK (1:1,000; cat. no. 4092; Cell Signaling Technology, Inc., Danvers, MA, USA), mouse monoclonal anti-p-JNK (1:1,000; cat. no. 9255; Cell Signaling Technology, Inc.) or rabbit monoclonal anti-p-ERK (1:1,000; cat. no. 9101; Cell Signaling Technology, Inc.). The blots were washed with TBST buffer and subsequently incubated with peroxidase-conjugated anti-mouse or anti-rabbit immunoglobulin G (1:2,000; cat. nos. sc-2030 or sc-2031; Santa Cruz Biotechnology, Inc., Santa Cruz, CA, USA). Following washing with TBST, the blots were developed with enhanced chemiluminescence reagents (Santa Cruz Biotechnology, Inc.). Rabbit polyclonal anti-β-actin antibody (1:500; cat. no. sc-13065; Santa Cruz Biotechnology, Inc.) was used as a control for each sample.

### Statistical analysis

The results are presented as the mean ± standard deviation. The data were analyzed using a one-way analysis of variance and a post hoc multiple-comparison test for comparisons of the mean values among multiple treatment groups. P<0.05 was considered to indicate a statistically significant difference.

## Results

### MVA promotes HMC proliferation

**Figure 1:**
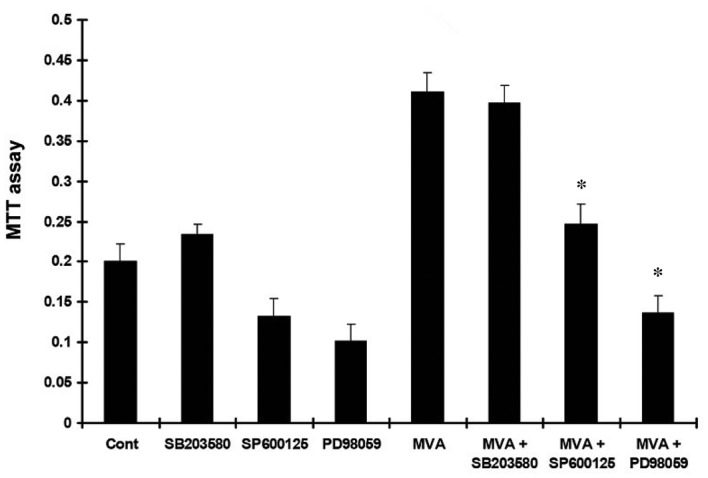
Results of the MTT reduction assay revealed that MVA significantly promoted the proliferation of HMCs (P<0.05, compared with the control). PD98059 and SP600125 significantly inhibited HMC proliferation (^*^P<0.05, vs. MVA only), while no significant effect on HMC proliferation was observed following treatment with SB203580. The results are presented as the mean ± standard deviation.. HMC, human mesangial cell; MVA, mevalonate; Cont, control.

**Figure 2:**
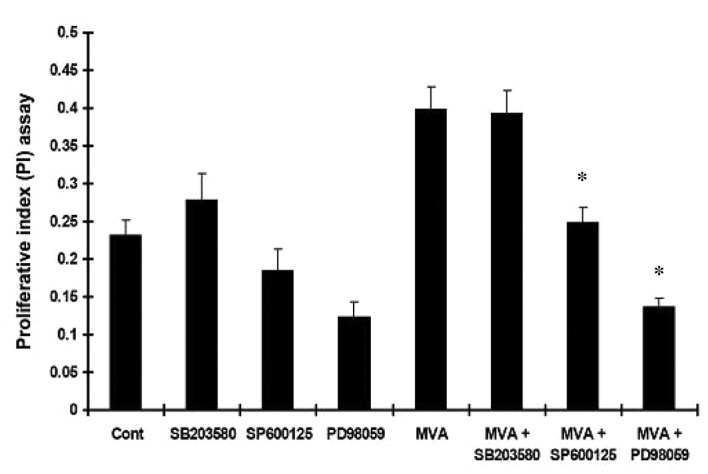
MVA treatment induced a significant increase in PI (P<0.05, compared with the control). PD98059 and SP600125 inhibited the increase in PI (^*^P<0.05, vs. MVA only), while no significant effect on PI was observed following treatment with SB203580. The results are presented as the mean ± standard deviation. PI, proliferative index; MVA, mevalonate; Cont, control.

To elucidate the role of MVA in HMC proliferation, the cultured HMCs were treated with MVA at various concentrations for 24 h, or for various durations. The results demonstrated that MVA produced a significant dose-dependent increase in the PI at concentrations <10^−7^ M, compared with the control (P<0.05). At concentrations of MVA >10^−7^ M, the PI was significantly decreased. MVA treatment also promoted HMC proliferation in a time-dependent manner (P<0.05; Tables I and II). Following culture of the HMCs with MVA in the presence or absence of PD98059, SP600125 or SB203580, the increased MTT absorbance (Fig. 1) and PI (Fig. 2) in the HMCs were inhibited by PD98059 and SP600125, but not by SB203580.

### MVA stimulates the expression of ECM proteins

**Figure 3:**
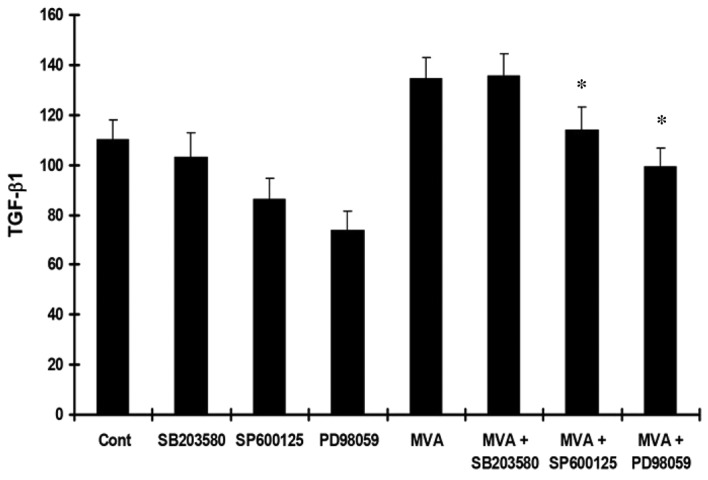
MVA treatment significantly increased the expression of TGF-β1 (P<0.05, compared with the control). PD98059 and SP600125 inhibited the expression of TGF-β1 (^*^P<0.05, vs. MVA only), while no significant effect on TGF-β1 was observed following treatment with SB203580. The results are presented as the mean ± standard deviation. MVA, mevalonate; Cont, control; TGF, tansforming growth factor.

**Figure 4:**
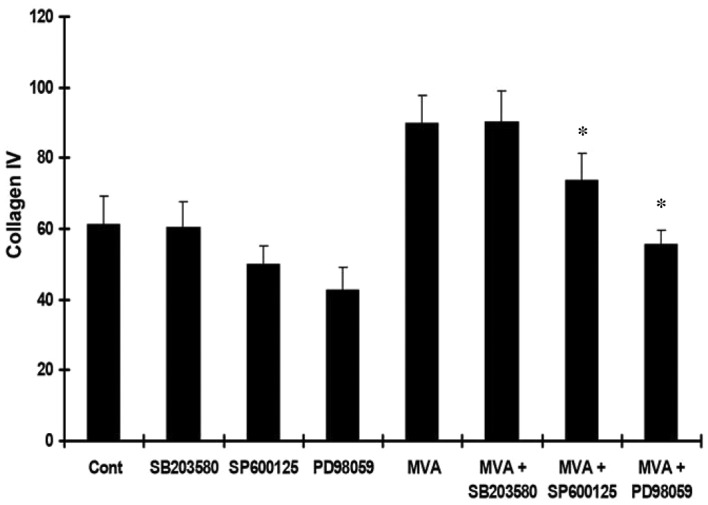
MVA treatment significantly promoted the expression of Col-IV (P<0.05, vs. control). PD98059 and SP600125 inhibited the secretion of Col-IV (^*^P<0.05, vs. MVA only), while no significant effect on Col-IV was observed following treatment with SB203580. The results are presented as the mean ± standard deviation. MVA, mevalonate; Cont, control; Col-IV, collagen type IV.

**Figure 5:**
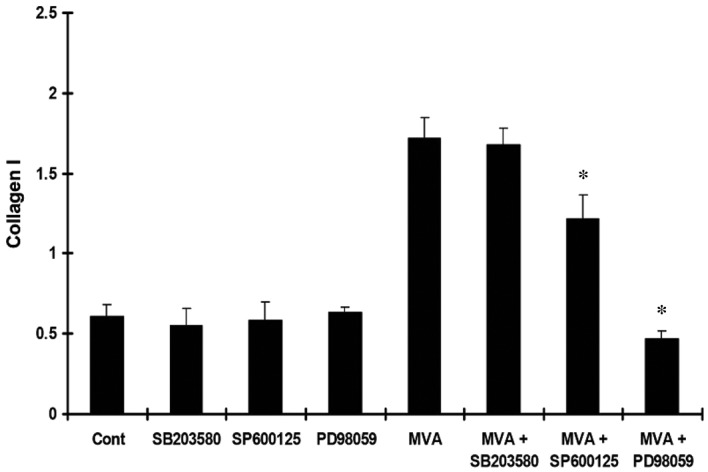
Treatment with MVA significantly promoted the expression of Col-I (P<0.05, vs. control). PD98059 and SP600125 inhibited the expression of Col-I (^*^P<0.05, vs. MVA only), while no significant effects were observed on the expression of Col-I following treatment with SB203580. The results are presented as the mean ± standard deviation. MVA, mevalonate; Cont, control; Col-I, collagen type I.

The HMCs were cultured with MVA alone or in the presence of PD98059, SP600125 or SB203580. An ELISA was performed to determine the levels of TGF-β1, Col-IV and Col-I. Treatment with MVA significantly increased the expression of TGF-β1 (Fig. 3), Col-IV (Fig. 4) and Col-I (Fig. 5). However, the effects were reversed by PD98059 and SP600125, while no significant effect was observed following treatment with SB203580.

### MVA suppresses apoptotic signaling in the HMCs

**Figure 6:**
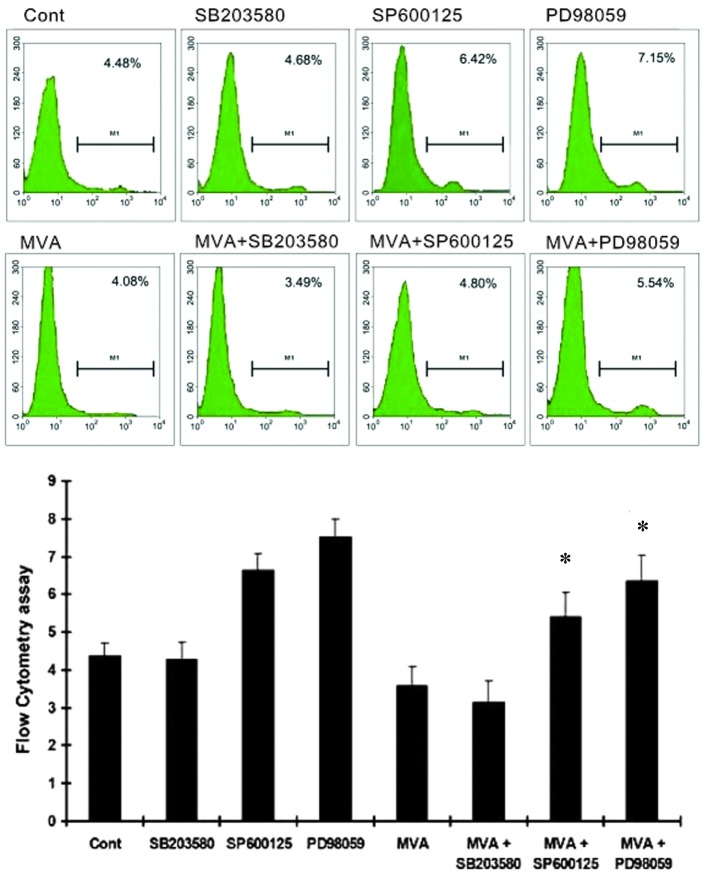
MVA treatment significantly inhibited human mesangial cell apoptosis (P<0.05, vs. control). PD98059 and SP600125 markedly increased the number of apoptotic cells (^*^P<0.05, vs. MVA only), while no effect was observed following treatment with SB203580. The results are presented as the mean ± standard deviation. MVA, mevalonate; Cont, control.

**Figure 7:**
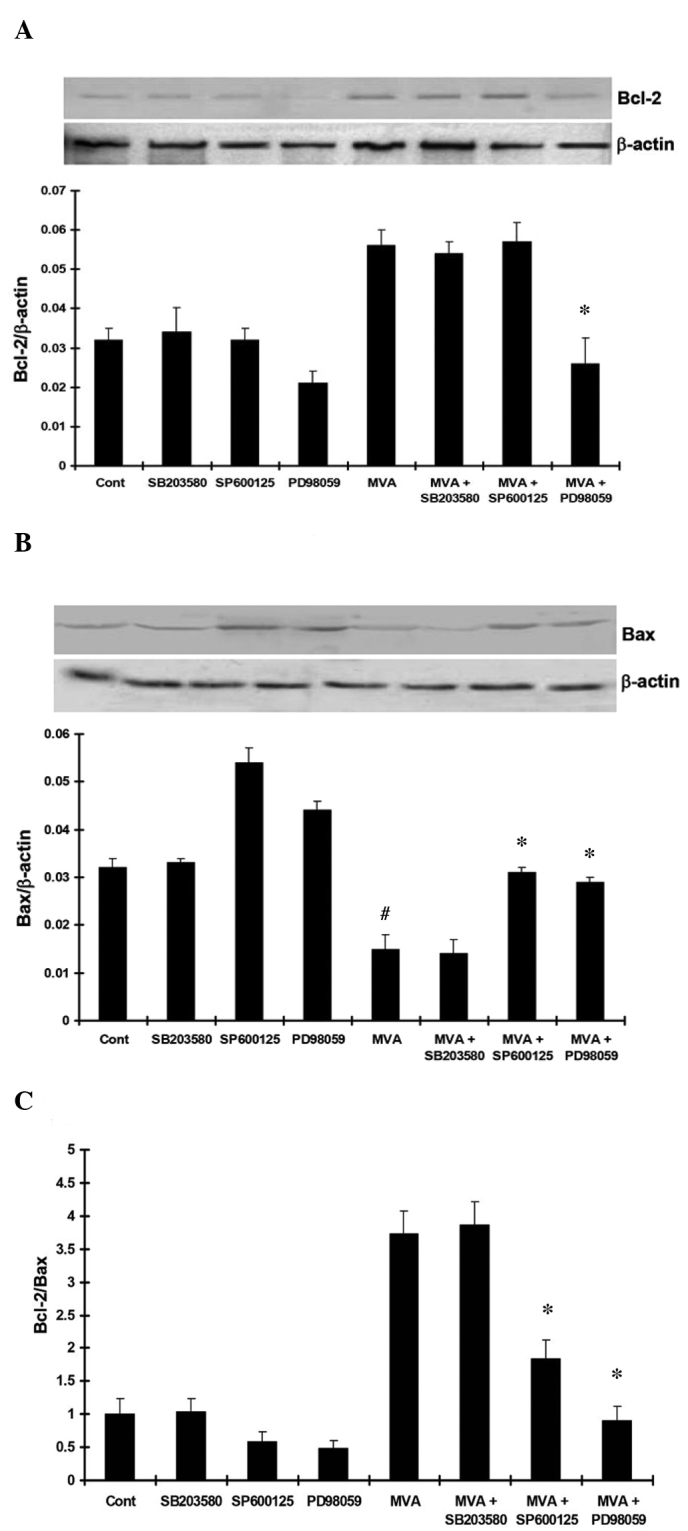
(A) MVA treatment significantly upregulated the expression of Bcl-2 (P<0.05, vs. control). PD98059 and SP600125 inhibited the upregulation of Bcl-2, while no effect was observed following treatment with SB203580. (B) MVA significantly downregulated the expression of Bax (P<0.05, vs. control). PD98059 and SP600125 inhibited the downregulation of Bax, while no effect was observed following treatment with SB203580. (C) MVA significantly upregulated the Bcl-2/Bax ratio (P<0.05, vs. control). PD98059 and SP600125 inhibited the upregulation of the Bcl-2/Bax ratio, while no effect was observed following treatment with SB203580. The results are presented as the mean ± standard deviation. ^*^P<0.05, vs. MVA only; ^#^P<0.05, vs. the control. MVA, mevalonate; Cont, control; Bcl-2, B-cell lymphoma 2; Bax, Bcl-2-associated X protein.

To elucidate the effect of MVA on HMC apoptosis, the HMCs were cultured with MVA alone or in the presence of PD98059, SP600125 or SB203580. Flow cytometric analysis was performed to assess HMC apoptosis. The expression levels of Bcl-2 and Bax were analyzed using western blot analysis. The data revealed that MVA treatment significantly inhibited HMC apoptosis. PD98059 and SP600125 markedly increased the number of apoptotic cells, while SB203580 had no effect (Fig. 6). In addition, the expression of Bcl-2 was significantly increased following treatment with MVA. PD98059 and SP600125 inhibited the upregulation of Bcl-2, while SB203580 had no effect on the expression of Bcl-2 (Fig. 7A). The expression of Bax was significantly downregulated by MVA treatment, and PD98059 and SP600125 inhibited this downregulation, No significant effect was observed following treatment with SB203580 (Fig. 7B). The ratio of Bcl-2/Bax was significantly increased following MVA treatment, however, this increased Bcl-2/Bax ratio was inhibited by treatment with PD98059 and SP600125, while no significant effect was observed following treatment with SB203580 (Fig. 7C).

### JNK and ERK mediate MVA-induced HMC proliferation

**Figure 8:**
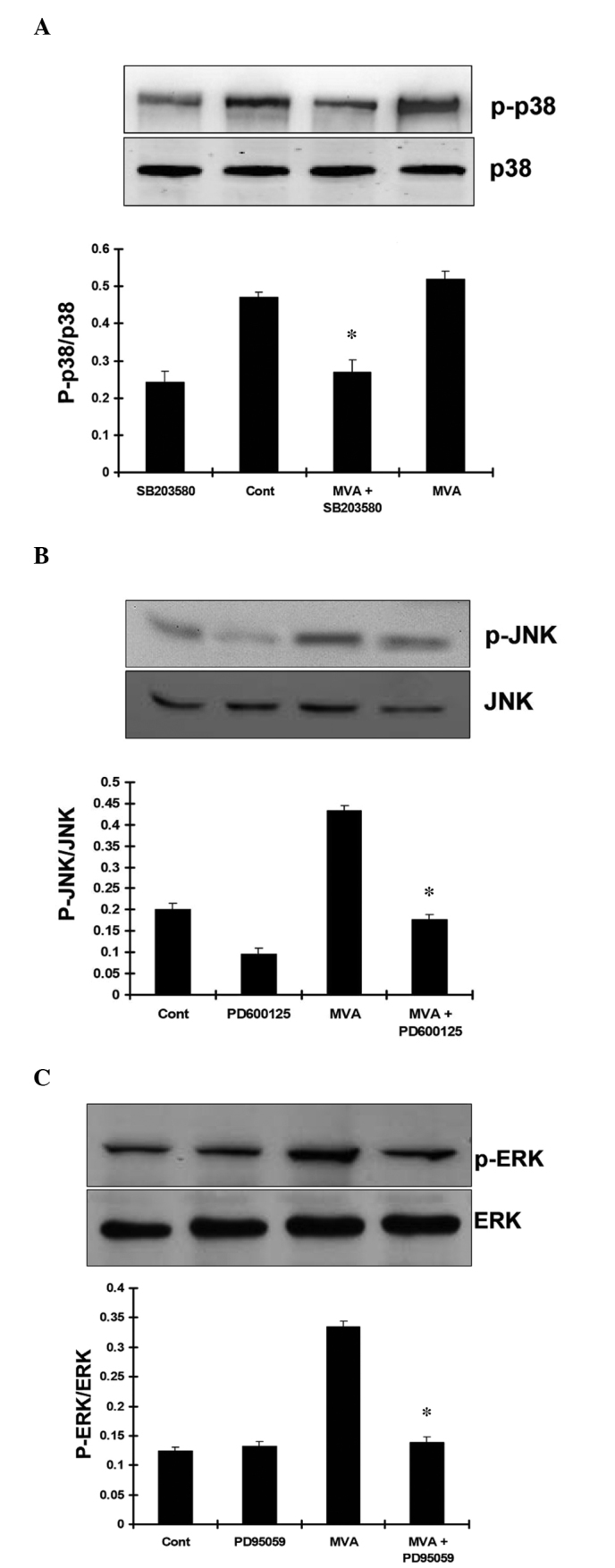
(A) MVA had no significant effect on the expression of p-p38 MAPK. SB203580 markedly inhibited the expression of p-p38 MAPK. (B) MVA significantly upregulated the expression of p-JNK (P<0.05, vs. control). SP600125 markedly inhibited the expression of p-JNK. (C) MVA significantly upregulated p-ERK expression (P<0.05, vs. control). PD98059 markedly inhibited the expression of p-ERK. The results are presented as the mean ± standard deviation ^*^P<0.05, vs. MVA only. MVA, mevalonate; Cont, control; MAPK, mitogen-activated protein kinase; ERK, extracellular signal-regulated kinase; JNK, c-Jun NH2-teminal kinase.

The HMCs were cultured with MVA alone or in the presence of PD98059, SP600125 and SB203580. Western blot analysis was performed to analyze the protein expression levels of p-ERK1/2, p-JNK and p-p38. The results demonstrated that treatment with MVA had no significant effect on the expression of p-p38. Treatment with SB203580 markedly inhibited the expression of p-p38 MAPK (Fig. 8A). However, MVA significantly stimulated the expression levels of JNK (Fig. 8B) and ERK (Fig. 8C). As expected, SP600125 and PD98059 markedly inhibited the phosphorylation of JNK and ERK, respectively.

## Discussion

MC proliferation and ECM deposition are important in renal fibrosis (9). Emerging evidence has demonstrated that hyperlipidemia, particularly of low-density lipoprotein, is involved in the proliferation of MCs, and HMG-CoA inhibitors can inhibit this process (10). It has been suggested that the HMG-CoA inhibitors inhibit this process by inhibiting MVA metabolism. Thus, the present study hypothesized that MVA promotes MC proliferation and mesangial matrix deposition. The results of the present study demonstrated that MVA promoted HMC proliferation in a dose-dependent manner (1×10^−9^–1×10^−7^ mol/l), with maximal stimulatory effects at a concentration of 1×10^−7^. However, higher concentrations (>1×10^−6^ mol/l) inhibited HMC proliferation, which may be due to higher concentrations exerting cytotoxic effects and inhibiting HMC proliferation. The PI is an index reflecting DNA synthesis. The results revealed that MVA increased the PI in the dose-dependent manner, which coincided with its stimulatory effects on MC proliferation.

Subsequently, the mechanisms of MVA on HMC proliferation and ECM deposition were investigated. TGF-β is a major factor in stimulating the expression of fibrotic protein, and it has been observed that TGF-β promotes ECM accumulation (11,12). *In vitro*, TGF-β promotes MC proliferation and matrix synthesis. The present study demonstrated that MVA treatment promoted the expression of TGF-β1 and thus promoted the expression of ECM-associated proteins, including Col-IV and Col-I. These responses may contribute to glomerular sclerosis and fibrosis.

Dysregulation of apoptotic pathways may also contribute to renal damage and fibrosis (13). Bcl-2 family members, including Bcl-2, an anti-apoptotic protein, and Bax, a pro-apoptotic protein, tightly regulate cell apoptosis. The ratio of Bcl-2 to Bax determines the survival of the cells. The present study revealed that MVA treatment upregulated the expression of Bcl-2, while downregulating the expression of Bax, which increased the Bcl-2/Bax ratio and inhibited HMC apoptosis. This may have contributed to the increase in the number of HMCs and ECM accumulation.

The molecular mechanisms underlying the effects MVA on the HMCs were investigated. MAPKs, including ERK, JNK/SAPK, P38 MAPK and ERK5/BMK1, are key regulators of MC proliferation and ECM deposition and are thus closely associated with the development of mesangial proliferative glomerulonephritis. In the present study, the MAPKs were investigated using their specific inhibitors. The results demonstrated that MVA treatment significantly stimulated JNK MAPK. SP600125, a specific inhibitor of JNK MAPK, markedly promoted the expression of Bax and inhibited the Bcl-2/Bax ratio. However, SP600125 had no significant effect on the PI. These findings indicated that JNK MAPK may promote HMC proliferation by downregulating the expression of Bax, thus inhibiting HMC apoptosis. As SP600125 had no significant effect on the expression levels of TGF-β1, Col-IV or Col-I, it was hypothesized that the effects of JNK on HMCs may be secondary to its inhibitory effects on cell apoptosis.

The present study also demonstrated that PD98059, a specific ERK inhibitor, promoted HMC apoptosis, inhibited the expression levels of TGF-β1, Col-IV, Col-I and Bcl-2, and upregulated the expression of Bax, therefore, decreasing the Bcl-2/Bax ratio. These findings coincided with the finding that MVA promoted the phosphorylation of ERK. These results indicated that MVA promoted the expression of TGF-β1 in an autocrine manner, resulting in HMC proliferation and ECM accumulation. By contrast, treatment with MVA inhibited HMC apoptosis by regulating the expression levels of Bcl-2 and Bax.

P38 MAPK is another important component of the MAPK family. In the present study SB203580, a specific inhibitor of p38 MAPK, exhibited no effects on HMC proliferation, cell apoptosis or the expression levels of TGF-β1 Bcl-2 or Bax, indicating that the effects of MVA on the HMCs were not mediated by the P38 MAPK pathway.

Crosstalk among MAPKs is common in mammalian cells (14). In the present study, treatment with MVA activated JNK MAPK and ERK MAPK, but had no effect on p38 MAPK. Despite this, whether any crosstalk occurs between JNK MAPK and ERK MAPK remains to be elucidated (15,16). The association between different MAPKs and their potential crosstalk requires further investigation in HMCs.

In conclusion, the results of the present study revealed the mechanism by which MVA stimulates MC proliferation, and may provide a therapeutic strategy in the treatment of proliferative glomerular disease.

## Figures and Tables

**Table I:** Effects of different concentrations of MVA on human mesangial cell proliferation.

MVA concentration (mol/l)	Absorbance at 490 nm		
	12 h	24 h	48 h
0	0.119±0.024	0.206±0.015^a^	0.400±0.019^b^
1×10^−9^	0.153±0.047^c^	0.284±0.033^a^,^c^	0.409±0.017^b^,^c^
1×10^−8^	0.171±0.053^c^,^d^	0.302±0.041^a^,^c^,^d^	0.499±0.010^b^,^c^,^d^
1×10^−7^	0.201±0.040^c^,^e^	0.326±0.019^a^,^c^,^e^	0.509±0.011^b^,^c^,^e^
1×10^−6^	0.184±0.060^c^,^f^	0.317±0.016^c^,^a^,^f^	0.406±0.007^b^,^f^
1×10^−5^	0.149±0.028^c^,^g^	0.263±0.029^c^,^a^,^g^	0.390±0.010^b^,^g^
1×10^−4^	0.121±0.025^h^	0.201±0.031^a^,^h^	0.306±0.005^b^,^h^
1×10^−3^	0.088±0.015^i^	0.175±0.027^a^,^i^	0.301±0.011^b^,^i^

Data are expressed as the mean ±standard deviation (n=3).

a P<0.05 vs. 12 h;

b P<0.05 vs. 24 h.

c P<0.05 vs. 0 mol/l,

d P<0.05 vs. 1×10^−9^ mol/l,

e P<0.05 vs. 1×10^−8^ mol/l,

f P<0.05 vs. 1×10^−7^ mol/l,

g P<0.05 vs. 1×10^−6^ mol/l,

h P<0.05 vs. 1×10^−5^ mol/l,

i P<0.05 vs. 1×10^−4^ mol/l. MVA, mevalonate.

**Table II:** Effects of different concentrations of MVA on proliferative index.

MVA concentration (mol/l)	Proliferation index		
	12 h	24 h	48 h
0	14.3±3.4	19.1±2.6^a^	25.5±2.8^b^
1×10^−9^	21.9±2.2^c^	25.1±3.3^a^,^c^	31.9±3.6^b^,^c^
1×10^−8^	23.2±2.0^c^,^d^	27.1±.2.6^a^,^c^,^d^	33.9±3.8^b^,^c^,^d^
1×10^−7^	27.1±3.14^c^,^e^	31.2±3.2^a^,^c^,^e^	38.3±4.4^b^,^c^,^e^
1×10^−6^	19.7±2.1^c^,^f^	23.4±1.6^c^,^a^,^f^	29.5±2.5^b^,^f^
1×10^−5^	17.1±2.2^c^,^g^	20.5±2.7^a^,^g^	25.8±1.6^b^,^g^
1×10^−4^	14.6±1.5^h^	17.4±2.0^a^,^h^	21.6±1.5^b^,^h^
1×10^−3^	12.7±1.2^i^	14.5±2.1^a^,^i^	17.3±1.3^b^,^i^

Data are expressed as the mean ±standard deviation (n=3).

a P<0.05 vs. 12 h;

b P<0.05, vs. 24 h.

c P<0.05, vs. 0 mol/l,

d P<0.05, vs. 1×10^−9^ mol/l,

e P<0.05, vs. 1×10^−8^ mol/l,

f P<0.05, vs. 1×10^−7^ mol/l,

g P<0.05, vs. 1×10^−6^ mol/l,

h P<0.05, vs. 1×10^−5^ mol/l,

i P<0.05,=, vs. 1×10^−4^ mol/l. MVA, mevalonate.
